# Programmed cell death-ligand 1 expression predicts survival in patients with gastric carcinoma with microsatellite instability

**DOI:** 10.18632/oncotarget.14519

**Published:** 2017-01-05

**Authors:** Junhun Cho, Jeeyun Lee, Heejin Bang, Seung Tae Kim, Se Hoon Park, Ji Yeong An, Min Gew Choi, Joon Ho Lee, Tae Sung Sohn, Jae Moon Bae, Won Ki Kang, Sung Kim, Kyoung-Mee Kim

**Affiliations:** ^1^ Department of Pathology, Soonchunhyang University Cheonan Hospital, Soonchunhyang University College of Medicine, Cheonan, Korea; ^2^ Department of Medicine, Division of Hematology-Oncology, Samsung Medical Center, Sungkyunkwan University School of Medicine, Seoul, Korea; ^3^ Department of Pathology and Translational Genomics, Samsung Medical Center, Sungkyunkwan University School of Medicine, Seoul, Korea; ^4^ Department of Surgery, Samsung Medical Center, Sungkyunkwan University School of Medicine, Seoul, Korea

**Keywords:** programmed death-ligand 1, gastric cancer, prognosis, immune, therapy

## Abstract

Programmed death-ligand 1 (PD-L1) is expressed in a subgroup of gastric cancers that may benefit from immunotherapy. Microsatellite instability-high (MSI-H) is a potential predictive factor for response to immunotherapy targeting the PD-1 or its ligand PD-L1. The relationship between PD-L1 expression and MSI-H status remains poorly understood. In this study, we investigated PD-L1 expression in patients with MSI-H gastric cancer. We analyzed PD-L1 expression in 78 MSI-H gastric cancer tissue samples using immunohistochemistry. PD-L1 expression was classified as expression on tumor cells or on immune cells. We observed PD-L1 expression in 48 gastric cancer samples (61.5%), consisting of 7 (9.0%) cases with tumor PD-L1 expression and 47 (60.3%) cases with immune cell PD-L1 expression. Immune cell PD-L1 expression was frequently associated with intestinal type cancer by the Lauren classification (*p* = 0.015), with a lower risk of lymph node metastasis (*p* = 0.027) and lower tumor stages (*p* = 0.029) compared to MSI-H gastric cancers without PD-L1 expression. Moreover, immune cell PD-L1 expression was an independent favorable prognostic factor for overall survival (versus PD-L1 negative; hazard ratio, 3.451; 95% confidence interval, 1.172–12.745; *p* = 0.025). In MSI-H gastric cancer, PD-L1 expression was observed to be independently associated with a longer survival.

## INTRODUCTION

Gastric cancer is the fourth most commonly diagnosed cancer and the second leading cause of cancer-related mortality worldwide [1]. The most effective treatment for localized gastric carcinoma is curative resection; however, approximately half of all patients with advanced-stage disease experience recurrence following surgery. The postoperative prognosis of patients with advanced gastric carcinoma remains poor [2, 3].

The Cancer Genome Atlas (TCGA) research network proposed a molecular classification dividing gastric carcinoma into four subtypes. One of them, microsatellite instability-high (MSI-H) tumors, is associated with elevated mutation rates including mutations in genes encoding targetable oncogenic signaling proteins [4]. The rate of incidence of MSI-H status in gastric carcinoma was previously reported to be 8.5%–37.8% [5]. However, the prognostic significance of MSI-H status in gastric carcinoma has been controversial [6–11]. Recently, a hypothesis has been proposed that cancers with a high prevalence of somatic mutations due to mismatch-repair defects may be susceptible to immune checkpoint blockade [12]. A phase 2 clinical trial for evaluating the clinical activity of pembrolizumab, the first programmed death 1 (PD-1) inhibitor, revealed that patients with MSI-H colorectal carcinomas and high somatic mutation loads are associated with better prognosis than patients with microsatellite stable (MSS) cancer [13]. Programmed death receptor-ligand 1 (PD-L1; also known as B7-H1 or CD274) is known to play an important role in immune evasion of tumor cells. PD-L1 on tumor cells interacts with PD-1 on T cells and decreases T cell receptor (TCR)-mediated proliferation and cytokine production [14]. Therefore, the inhibition of PD-1/PD-L1 interactions might improve the efficacy of adoptive cell therapy for malignancies. Previous studies have shown that the expression of PD-L1 is linked with a worse prognosis in patients with cancer compared with those without PD-L1 expression; however, this finding remains controversial in case of some cancers [15]. Multiple studies on gastric carcinoma utilizing immunohistochemistry (IHC) for PD-L1 demonstrated varying rates of PD-L1 expression, ranging from 5.1% to 65.0% [16–26]. In the majority of studies, PD-L1 expression was associated with an unfavorable prognosis [16–22, 25, 26]; however, recent studies have linked PD-L1 expression with a favorable prognosis [23, 24].

Although the relationship between MSI-H gastric carcinoma and PD-L1 status has not been fully explored, we hypothesized that a therapeutic strategy targeting the PD-1/PD-L1 interactions may be more effective in patients with MSI-H gastric carcinoma than in those with MSS gastric carcinoma. Accordingly, the connection between MSI-H status and PD-L1 expression in patients with gastric carcinoma requires investigation. In the present study, we investigated the clinicopathological characteristics of PD-L1 expression in patients with MSI-H gastric carcinoma and found that PD-L1 expression was an independent prognostic factor for the survival of such patients.

## RESULTS

### Patient characteristics and PD-L1 expression

The mean age of the patients with gastric carcinoma was 65.9 (range, 31−88). The male-to-female ratio was 1.6:1. The mean follow-up period was 56.9 ± 26.1 months. Eighteen (23.1%) patients experienced a recurrence, and 16 (20.5%) patients died of the disease. Of the 78 gastric cancer cases, 15 (19.2%) were diagnosed as stage I, 30 (38.5%) as stage II, 23 (29.5%) as stage III, and 10 (12.8%) as stage IV. All patients were Epstein-Barr virus (EBV)-negative.

**Figure 1:**
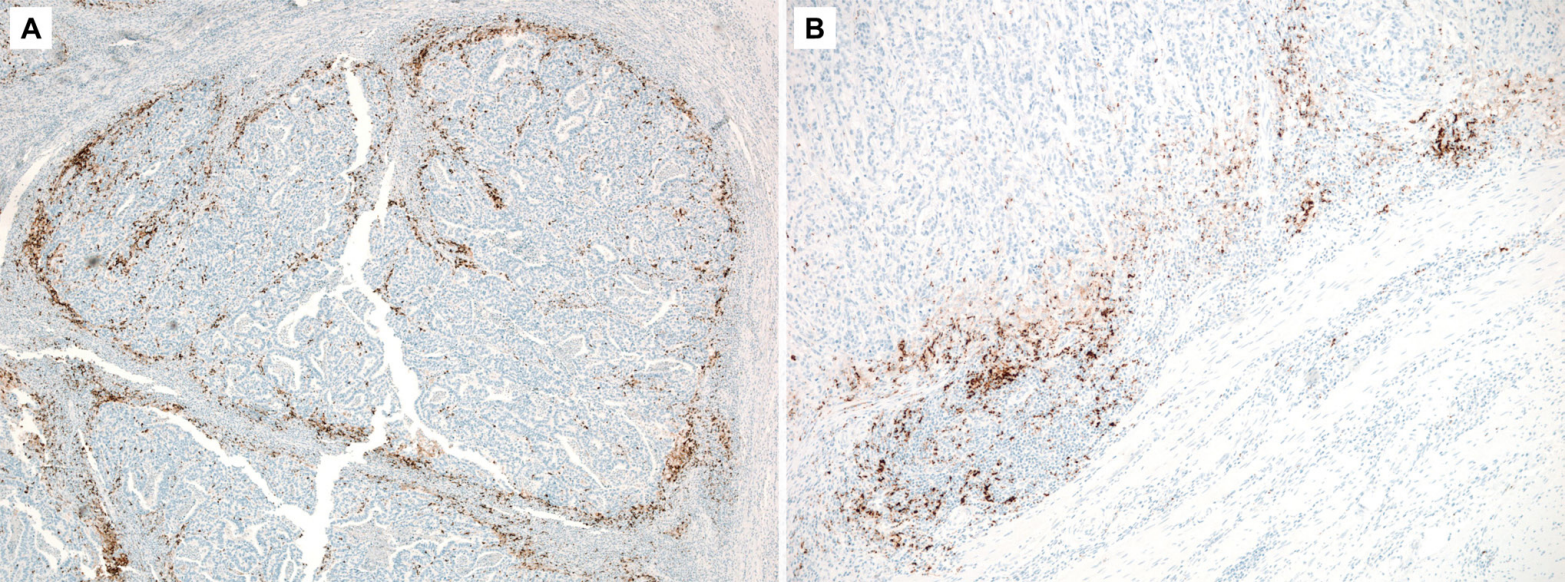
A representative photomicrograph of PD-L1 immunohistochemistry showing high staining in the deeply invasive front of the tumor

Prominent staining of PD-L1 was observed at the periphery of the tumor, i.e., the infiltrating front (Figure 1). Seven cases (9.0%) and 47 cases (60.3%) were classified into the groups showing PD-L1 expression in tumor cells (PD-L1^TC+^) and in immune cells (PD-L1^IC+^), respectively. Six of 7 patients with PD-L1^TC+^ status were also positive for PD-L1^IC+^ status. Table 1 summarizes the clinicopathological findings based on PD-L1 expression status. In MSI-H gastric carcinoma, PD-L1^IC+^ status was more frequently observed in intestinal type tumors (*p* = 0.015), as well as in those with a lower risk of lymph node metastasis (*p* = 0.027) and lower tumor, node, and metastasis (TNM) classification stages compared to PD- L1^IC−^ group (*p* = 0.029). However, there was no significant association between PD-L1^TC+^ status and histological type of the tumor by the Lauren classification, pN status, or the TNM stage classification. Age, sex, location, and pT status demonstrated no significant correlation with PD-L1 IHC in either tumor cells or immune cells.

**Table 1:** Demographic distribution and clinicopathological features in microsatellite instability-high gastric cancer

		Total	Tumor cells					Immune cells				
		**Negative**		**Positive**		***p* value**	**Negative**		**Positive**		***p* value**
		**Number (%)**		**Number (%)**			**Number (%)**		**Number (%)**		
Age	< 60	19	19	(100.0)	0	(0.0)	0.129	8	(42.1)	11	(57.9)	0.507
	≥ 60	59	52	(88.1)	7	(11.9)		23	(39.0)	36	(61.0)	
Sex	Male	48	43	(89.6)	5	(10.4)	0.449	20	(41.7)	28	(58.3)	0.422
	Female	30	28	(93.3)	2	(6.7)		11	(36.7)	19	(63.3)	
Location	Cardia	3	2	(66.7)	1	(33.3)	0.437	1	(33.3)	2	(66.7)	0.270
	Body	16	15	(93.8)	1	(6.3)		3	(18.8)	13	(81.3)	
	Antrum	55	50	(90.9)	5	(9.1)		25	(45.5)	30	(54.5)	
	Multiple/Whole	4	4	(100.0)	0	(0.0)		2	(50.0)	2	(50.0)	
Lauren type	Intestinal	48	44	(91.7)	4	(8.3)	0.671	16	(33.3)	32	(66.7)	0.015
	Diffuse	25	23	(92.0)	2	(8.0)		15	(60.0)	10	(40.0)	
	Mixed	5	4	(80.0)	1	(20.0)		0	(0.0)	5	(100.0)	
EBV	Negative	78	71	(91.0)	7	(9.0)		31	(39.7)	47	(60.3)	
	Positive	0	0		0			0		0		
pT status	pT1	1	1	(100.0)	0	(0.0)	0.595	0	(0.0)	1	(100.0)	0.054
	pT2	45	42	(93.3)	3	(6.7)		14	(31.1)	31	(68.9)	
	pT3	19	16	(84.2)	3	(15.8)		10	(52.6)	9	(47.4)	
	pT4	13	12	(92.3)	1	(7.7)		7	(53.8)	6	(46.2)	
pN status	pN0	16	15	(93.8)	1	(6.3)	0.562	2	(12.5)	14	(87.5)	0.027
	pN1	34	32	(94.1)	2	(5.9)		15	(44.1)	19	(55.9)	
	pN2	21	17	(81.0)	4	(19.0)		10	(47.6)	11	(52.4)	
	pN3	7	7	(100.0)	0	(0.0)		4	(57.1)	3	(42.9)	
TNM stage	I	15	15	(100.0)	0	(0.0)	0.313	2	(13.3)	13	(86.7)	0.037
	II	30	27	(90.0)	3	(10.0)		13	(43.3)	17	(56.7)	
	III	23	19	(82.6)	4	(17.4)		10	(43.5)	13	(56.5)	
	IV	10	10	(100.0)	0	(0.0)		6	(60.0)	4	(40.0)	

Abbreviations: EBV, Epstein-Barr virus.

### PD-L1 expression and prognosis

**Figure 2:**
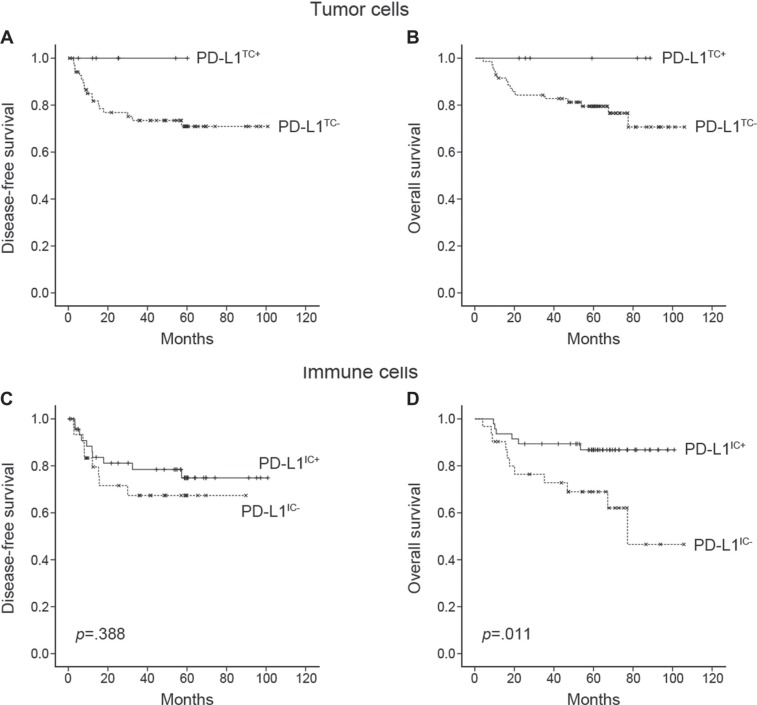
Kaplan-Meier survival curves of disease-free survival and overall survival according to PD-L1 expression in tumor cells (**A, B**) and immune cells (**C, D**). No statistics were computed in A and B because all PD-L1^TC+^ (*n* = 7) cases were censored.

The mean disease-free survival (DFS) and overall survival (OS) of patients with PD-L1^IC+^ gastric carcinoma was higher than that of patients with PD- L1^IC−^ gastric carcinoma (44.4 and 62.2 months vs. 33.9 and 48.7 months, respectively). PD-L1^IC+^ status was significantly associated with a longer OS (log rank *p* = 0.011) but not with a longer DFS (Figure 2). PD-L1 expression in tumor cells showed a trend towards a better prognosis; however, it was not significantly related to DFS or OS. All seven PD-L1^TC+^ patients are alive without disease recurrence during the follow-up period (mean OS, 55.7 months). Two of them were TNM stage II and five of them had TNM stage III disease. Of nine TNM stage IV patients, six patients died after recurrence and they were all PD-L1 negative. Other three patients survived without disease progression were unexceptionally PD-L1^IC+^ and their DFS were 63.8, 68.4, and 69.6 months, respectively. In multivariate analysis, the PD-L1^IC−^ group showed a significantly shorter OS (95% confidence intervals, 1.172– 10.162; hazard ratio, 3.451) when compared with the PD-L1^IC+^ group (Table 2).

**Table 2:** Multivariate analysis of death in all 78 patients with microsatellite instability-high gastric cancer

		HR	95% CI	*p* value
PD-L1 in IC				0.025
	PD-L1^IC+^	ref		
	PD-L1^IC−^	3.451	1.172–12.745	
TNM stage				0.030
	I	ref		
	II	1.384	0.150–12.745	
	III	2.597	0.293–23.020	
	IV	7.857	0.905–68.218	

Abbreviations: HR, hazard ratio; CI, confidence interval.

## DISCUSSION

Recently, anti-PD-1/PD-L1 antibodies have shown remarkable therapeutic effects in advanced solid cancers. However, the interaction between PD-L1 expression and MSI remains poorly understood. We investigated PD-L1 expression in patients with MSI-H gastric carcinoma and observed that PD-L1 was expressed in 61.5% of tumors and that it was an independent favorable prognostic factor for survival.

To establish an anti-PD-1/PD-L1 therapeutic strategy, it is important to explore the relationship between MSI-H gastric carcinoma and PD-L1 expression. Previous studies revealed an adverse prognostic effect of PD-L1 expression in various cancers [27–35]. However, several recent studies have reported that a higher PD- L1 expression is associated with an increased number of tumor-infiltrating lymphocytes (TILs) and a longer survival in metastatic melanoma [18], Merkel cell carcinoma [36], non-small cell lung cancer [37, 38], and breast cancer [39]. In gastric carcinoma, patients with PD- L1-positive cancer showed significantly shorter survivals compared with those with PD-L1 negative cancer [16–22, 25, 26]. However, recent studies have found PD-L1 expression to be a favorable prognostic marker in gastric cancer [23, 24]. In the present study with MSI-H gastric carcinomas, we observed PD-L1^TC+^ in 9.0% of cases and PD-L1^IC+^ in 60.3% of cases. The positive rate of PD-L1 in tumor cells (9.0%) is generally lower than previous studies on gastric cancers (5.1% to 65.0%) [16–26]. It can be caused by the used primary antibody and diagnostic criteria of PD-L1 expression. Out of 11 previous studies, only one study used the same antibody as ours (clone SP142; Ventana, Tucson, AZ). In that study [26], PD-L1 positive GC was 40.9% (56/137): the criterion of PD-L1 positivity was 5% in tumor cells and their study consists many EBV-positive gastric carcinomas with lymphoid stroma, which are strongly associated with PD-L1 expression in tumor cells.

Unexpectedly, intra- or peritumoral immune cells with PD-L1 expression was associated with a longer OS. All 7 patients with PD-L1^TC+^ status survived and none experienced recurrence during their long (mean: 55.7 months) follow-up period, which was interrupted to achieve statistical significance. Moreover, PD-L1 expression in TILs was observed in 85.7% of patients with a PD-L1^TC+^ status. This finding suggested that the expression of PD-L1 in tumor cells and intra- and peritumoral immune cells is closely related. Moreover, the release of interferon gamma (IFN-γ) by TILs can directly induce PD-L1 expression in tumor cells and immune cells [40–42].

Patients with gastric carcinoma and high TIL levels have been reported to have a favorable prognosis [7, 13, 43]. It has been reported that PD-1/PD-L1 interaction inhibits T lymphocyte proliferation, survival, and effector functions; promotes the differentiation of CD4+ T cells into Foxp3+ regulatory T (Treg) cells; and increases the resistance of tumor cells to cytotoxic T lymphocyte attacks [44]. However, we found a longer OS in patients with MSI-H gastric carcinoma with PD- L1 expression, an observation that was contradictory to the previous theory. However, the present results are consistent with our recent report that a higher number of Tregs among CD4+ T cells correlated with increased OS and DFS and it was an independent prognostic factor for gastric carcinoma [45]. PD-L1 expression in tumors can be driven either by constitutive oncogene activation or by inflammation-mediated immune attack [46]. According to the cancer immunoediting hypothesis [47], newly developing tumor cells are detected and destroyed by the innate and adaptive immune systems, the so-called “elimination phase”. Following this, tumor cells that survive the elimination phase enter the equilibrium phase. In this process, some tumor cells can acquire the ability to overcome the host antitumor immunity. One of the consequences of this process is PD-L1 upregulation. PD- L1 expression in tumor cells signifies not only immune evasion of the tumor, but also the preexistence of a robust antitumor immune attack. Immunoedited PD- L1- positive tumor cells cannot enter the escape phase and remain in the equilibrium phase; thus, the patient might experience a relatively indolent clinical course. This process is extremely complex and delicate, meaning that minimal diversity in genetic, racial, and environmental features of patients can result in various clinical outcomes. Taken together, the exact roles of PD-L1 in malignant tumors and anti-PD-1/PD-L1 antibodies in immunotherapy in patients with different cancers have not been well established yet. However, we assume that PD-L1 in gastric carcinoma may also have anti-tumorigenic properties through the fostering of particular immune reactions; the precise underlying mechanisms for the same will need to be elucidated in the near future.

Recent studies with a few MSI-H gastric carcinoma cases (indicated via IHC of mismatch repair proteins) demonstrated significantly higher rates of PD-L1 expression compared to MSS gastric carcinoma [24, 26, 48, 49]. Therefore, we studied a large number of MSI-H gastric cancers and the current research without direct comparison with MSS gastric cancers could be a limitation of the present study and warrants further study.

In a phase 1b clinical trial (KEYNOTE-012 study) conducted to assess pembrolizumab, 8 out of 36 patients (22.2%) with PD-L1-positive gastric carcinoma or gastroesophageal junction cancer were judged to have a partial response. Among 4 patients with MSI-H gastric carcinoma, 2 had a partial response and the other 2 had progressive disease [50]. One of the noteworthy findings of this trial is that the response rate for pembrolizumab was higher in the low PD-L1 tumor proportion score group and the high PD-L1 mononuclear inflammatory cell density score group than in the groups that had high and low scores, respectively, on these measures. These results support our findings that the expression of PD-L1 not only in tumor cells, but also in immune cells, is important in the antitumor microenvironment.

In summary, PD-L1 expression, especially in intra- and peritumoral immune cells, is related to longer survival in patients with MSI-H gastric carcinoma. Further studies are necessary to elucidate the underlying immunological pathways and to predict which gastric carcinoma subclass may respond most favorably to anti-PD-1/PD-L1 immunotherapy.

## MATERIALS AND METHODS

### Patient selection

Patients who underwent surgery for primary gastric carcinoma from September 2004 to September 2011 at Samsung Medical Center were eligible for this study. Among them, 78 patients with MSI-H tumors were selected from our previous study cohort of 620 patients including 58 (85%) cases from 68 MSI-H gastric cancers [15], 6 (100%) cases from ARTIST cohort [51] and 14 (100%) from SMC DASL study cohort [52].

All 78 patients underwent radical gastrectomy with D2 lymph node dissection with or without adjuvant chemoradiation therapy (INT-0116 regimen) [53]. No patient had any other uncontrolled cancer at the time of diagnosis of gastric carcinoma or during the follow-up period. Clinical data including demographic features, tumor characteristics, and treatment outcomes were obtained by reviewing medical records using the intranet resources of Samsung Medical Center. Tumor stage was defined according to the TNM classification described in the 7th edition of the AJCC cancer staging manual [54]. All patients provided informed consent according to our institutional guidelines.

### Microsatellite instability

For MSI analysis, we performed multiplex polymerase chain reaction (PCR) with five quasi-monomorphic mononucleotide repeat markers as previously described [55]. Briefly, genomic DNA was isolated from formalin-fixed, paraffin-embedded (FFPE) tumor samples with a QIAamp DNA mini kit (Qiagen, Valencia, CA, USA). Each sense primer was end-labeled with one of the fluorescent markers FAM, HEX, or NED. Amplified PCR products were run on an ABI Prism 3130 Genetic Analyzer (Applied Biosystems, Foster City, CA, USA). Allelic sizes were estimated by Genemapper 4.1 (Applied Biosystems), and samples with no allelic size variations in fewer than two of the microsatellites were classified as MSS. Tumors with allelic size variations in two or more of the microsatellite markers were considered MSI-H.

### EBV-encoded RNA *in situ* hybridization

Three-micrometer-thick sections were cut from each tissue block and mounted on Superfrost-plus slides (Thermo Scientific, Waltham, MA, USA). We performed *in situ* hybridization with BOND-MAX with an EBV-encoded RNA probe (Leica, Newcastle, UK). Only sections that showed a strong signal within almost all tumor cell nuclei were considered positive.

### Immunohistochemistry

**Figure 3:**
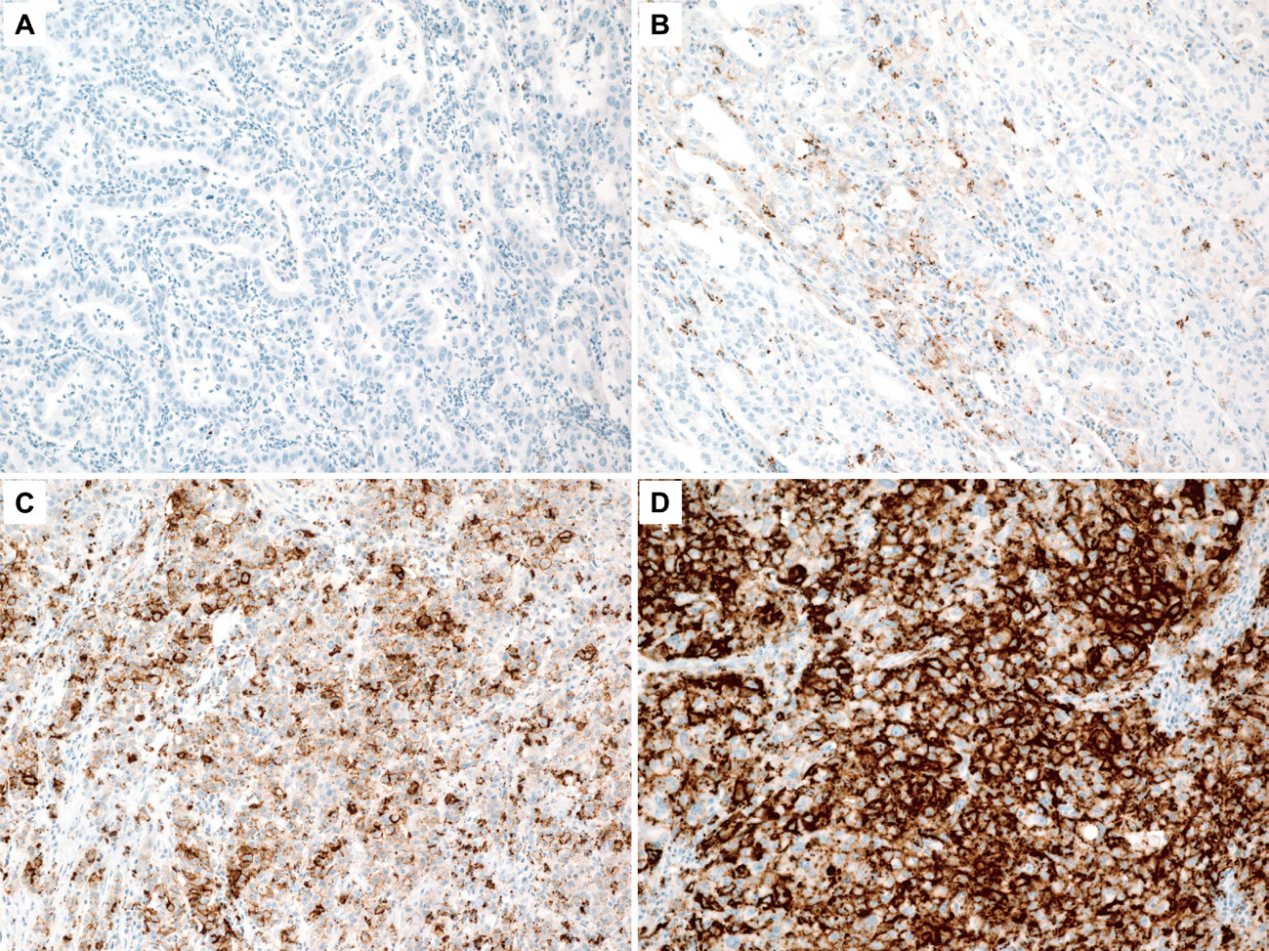
A tumor was scored as 0 (**A**), 1+ (**B**), 2+ (**C**), or 3+ (**D**) based on the intensity of cytoplasmic and/or membranous staining in > 10% of tumor cells.

We performed IHC on all 78 MSI-H gastric carcinoma samples. Staining for PD-L1 in FFPE tissue sections was conducted using a rabbit anti-human PD-L1 monoclonal antibody (clone SP142; Ventana, Tucson, AZ). The percentages of tumor cells and peritumoral immune cells that stained positive for PD-L1 were analyzed independently by two pathologists (J.C. and K.M.K.). A tumor was considered positive for PD-L1 if there was histological evidence of cytoplasmic and/or membranous staining. Percentages of PD-L1 positive tumor cells were quantified as 1%, 2%, 3 to 5%, 6 to 10%, and then in 10% increments up to 100% and the samples were given a score of 0, 1+, 2+, or 3+ based on the intensity of staining (Figure 3). Based on our preliminary statistical analyses and previous publication on solid cancers [56], scores of 2+ and 3+, and > 10% were defined as the criteria of PD- L1^TC+^. For peritumoral immune cells a score of 0, 1, 2, or 3 was given when < 1%; ≥ 1% but < 5%; ≥ 5% but < 10%; or ≥ 10% of cells per area were PD-L1-positive, respectively, as previously described [57]. Scores of 2 and 3 were classified as PD-L1^IC+^. To reduce interobserver variation, all the cases were reviewed by two pathologists, and in cases with disagreement, the final interpretation was determined by consensus using the multi-head microscope.

### Prognostic model building and statistical analysis

We analyzed the clinicopathological characteristics such as age, sex, pTNM stage (AJCC 7th edition), DFS, and OS of patients. For statistical analyses, we used SPSS 18.0 statistical software program (SPSS Inc., Chicago, IL, USA). We compared PD-L1 expression and clinicopathological variables with Pearson's chi-square test and the chi-square test using linear-by-linear association. We used the Kaplan-Meier method to estimate DFS and OS. To evaluate the association between clinicopathological factors and survival, the Cox proportional hazard model was used. *p*-values less than 0.05 were considered statistically significant.
